# Factor Structure and Equivalence of Maternal Resources for Care in Bangladesh, Vietnam, and Ethiopia

**DOI:** 10.1007/s10995-020-03100-4

**Published:** 2021-02-25

**Authors:** Sulochana Basnet, Edward A. Frongillo, Phuong Hong Nguyen, Spencer Moore, Mandana Arabi

**Affiliations:** 1 Department of Health Promotion, Education, and Behavior, University of South Carolina, 915 Greene Street, Discovery I, Columbia, SC 29208-4005 USA; 2 International Food Policy Research Institute, Washington, DC USA; 3 Global Technical Services, Nutrition International, Ottawa, ON Canada

**Keywords:** Resources for care, Caregiver, Woman, Children, Developing countries

## Abstract

**Objectives:**

Resources for care among women are crucial for children’s growth and development. The objectives of this cross-sectional study were to determine if: (1) the factor structure of measures of maternal resources for care was comparable across countries and consistent with the theoretical constructs and (2) the measures showed equivalence across contexts.

**Methods:**

The study included 4400, 4029 and 2746 women from Bangladesh, Vietnam, and Ethiopia, respectively. The measures of resources for care were maternal education, knowledge, height, body mass index, mental well-being, financial autonomy, decision-making, employment, support in chores, and perceived support.

**Results:**

The factor analysis demonstrated that a two-factor solution best explained the structure of resources for care in all three countries. The first factor was associated with financial autonomy and employment in all three countries and with decision-making in two countries. The second factor was associated with education and knowledge in all three countries. The measures of resources for care had measurement equivalence across countries.

**Conclusion for Practice:**

Resources for care were structurally similar and measurement equivalent across countries and can be used for measurement in low- and middle-income countries. Additional work examining the structure and cross-context equivalence of resources for care in other settings is warranted.

## Significance Statement

Resources for care available to women may play an important role in improving health and well-being of children. This study examined structure and equivalence of maternal resources for care in Bangladesh, Vietnam, and Ethiopia. Knowledge on structure and equivalence of maternal resources for care helps in the assessment and meaningful comparisons of the resources for care across various settings.

## Introduction

Despite attention from international communities and inclusion on the global agenda, poor child growth and development remain significant problems especially in low- and middle-income countries (Britto et al. 2017). Among several determinants of child growth and development, appropriate care is critical (Britto et al. 2017; Engle et al. 1999). Unavailability or inadequacy of resources available to caregivers can constrain provision of appropriate care (Engle et al. 1999).

Resources enhance ability of the individuals to make life choices and equip them for providing time, attention, and support to meet various needs of the household members, including children (Engle et al. 1999). The extended model of care of the United Nations Children’s Fund (UNICEF) highlights that care behaviors and child outcomes depend on resources for care, i.e., the capacity and ability of caregivers and families to provide care (Engle et al. 1999). The model identified six theoretical constructs for resources for care: education and knowledge, physical health, mental health, autonomy, reasonable workload and availability of time, and social support (Engle et al. 1999). Caregiver’s education and knowledge provide ability to provide care through processing of information, acquisition of skills, and modeling behavior. Caregiver’s health helps to transform acquired knowledge into practice (Engle et al. 1999). Autonomy reflects ability of caregivers to make decisions and control resources in household and community (Carlson et al. 2015; Engle et al. 1999). Reasonable workload and social support reflect family- and community-level resources that help strengthen caregiver’s ability to provide care (Engle et al. 1999).

Socio-cultural and ecological systems theories assert the critical role of caregivers and social environment on children’s development and well-being (Brofenbrenner 1994; Vygotsky 1978). Ecological systems theory indicates that children’s development depends on the environment in which children grow, including the people in the surrounding (Brofenbrenner 1994). Socio-cultural theory highlights the impact of caregivers, society, and culture on children’s life (Vygotsky 1978). Resources for caregivers are important to identify and address children’s needs, and the national, community, and household contexts could drive availability of the resources (Britto et al. 2017; Engle et al. 1999).

Women are children’s primary caregivers in almost all socio-cultural settings globally (Coller and Kuo 2015). Typically, mothers create the first environment to which children are exposed after birth. Children also spend most of the time during the early period of life with their family, especially mothers (Coller and Kuo 2015; Frongillo et al. 2017). The caregiver’s role in children’s well-being is greater in settings where accessibility of institutions or programs that promote nurturing care (e.g., preschool programs) is low (Borisova et al. 2017). Many programs that are focused on improving child health are operated through women, and women with less resources may not be able to take advantage of those programs (Tripathy et al. 2010). Therefore, building capacity and ability among caregivers, particularly women, could improve children’s well-being (Borisova et al. 2017; Engle et al. 1999).

Several ways of assessing the theoretical constructs of resources of care have been used. For example, education can be measured by years of schooling or literacy depending on the context. Data on education can also be collected using various methods like self-report and school records (Engle 1999). Some measures of resources for care are theoretically related, whereas other resources for care are distinct (Engle et al. 1999). Additionally, some types of resources for care can be measured objectively (e.g., height) or have been validated across many cultures (e.g., assessment of mental well-being) (WHO 1994). Some measures may not have the same meaning across cultures which may reduce their applicability (Engle et al. 1999; Taylor et al. 2004). Equivalence denotes that measures are consistent across contexts (Frongillo et al. 2019). Demonstration of the equivalence of measures across countries is essential to compare the research findings across contexts (Kankaraš and Moors 2010).

Understanding the structure and equivalence of the measures of resources for care may improve assessment and help in meaningful comparisons of the findings across cultures (Kankaraš and Moors 2010). Additionally, integrating interventions to promote capabilities of women could be cost-effective in improving child outcomes. Understanding the structure and equivalence of measures of resources for care may also help to develop and implement integrated interventions (Yousafzai et al. 2018). The objectives of the study were to determine if: (1) the factor structure of measures of maternal resources for care were comparable across countries consistent with the theoretical constructs and (2) the measures of resources for care showed equivalence across contexts. We hypothesized that factor structure of measures of resources for care would be comparable across countries and consistent with the theoretical constructs. We also hypothesized that the measures of resources for care will show equivalence across contexts.

## Methods

### Participants and Study Design

This cross-sectional study used the de-identified baseline data from household surveys collected in Bangladesh, Vietnam, and Ethiopia as a part of impact evaluation of the *Alive & Thrive* initiative in 2010. *Alive & Thrive* aims to increase survival, prevent illnesses, and support optimal growth and development of children by improving child feeding practices (Nguyen et al. 2017). Women and their children under the age of five years were included in the surveys (Bangladesh: 0–47.9 months, Vietnam and Ethiopia: 0–59.9 months). For this study, we used data pertaining to the women of youngest or index children only. The study samples were 4400, 4029, and 2746 women from Bangladesh, Vietnam, and Ethiopia, respectively. The sample sizes for factor analyses were slightly lower in all three countries (Bangladesh: 4396, Vietnam: 4022, and Ethiopia: 2740) because of missing data in a few cases for some variables.

### Data Collection

Informed consent for participation was obtained from the women prior to the inclusion in the study. Data were collected using structured interviews and anthropometry. Trainings were provided to the data collectors to improve data quality and reduce bias. The ethical approval for the data collection was obtained from the institutional review board of each country and the International Food Policy Research Institute. Detailed description of the data collection procedure can be found elsewhere (Ali et al. 2011; Nguyen et al. 2010; Saha et al. 2011).

### Measures

The data on resources for care were specific to the mothers of the children less than 5 years of age. The measures of resources for care used were maternal education, knowledge, physical health, mental well-being, financial autonomy, decision-making autonomy, employment, support in chores, and perceived instrumental support. No measure was available for the theoretical construct of reasonable workload. Information on all variables was reported by the participants (women), except the information pertaining to physical health which was based on the anthropometry.

#### Education and Knowledge

Years of schooling was used to represent educational attainment. Knowledge of the women was with regard to breastfeeding, complementary feeding, food diversity, iron deficiency symptoms, vitamin A sources, iodine fortification, and hand washing. The variable was developed by assigning a point for each correct answer, with a total possible score of 22 for Bangladesh, and 23 for Vietnam and Ethiopia.

#### Physical Health

Height and body mass index (kg/m^2^) were the measures of physical health. Heights and weights were measured by using height boards and electronic scales, respectively (Ali et al. 2011; Nguyen et al. 2010; Saha et al. 2011). Woman’s height and weight were measured twice by trained personnel and a third measurement was taken if the difference between two measurements was significant. The average of the measurements was used (Nguyen et al. 2010).

#### Mental Well-Being

Mental well-being was measured by using the Self-Reporting Questionnaire-20. The questionnaire has been validated and is considered reliable for use in low- and middle-income countries including our study settings (WHO 1994). The questionnaire includes twenty items on psychological and somatic symptoms over the past four weeks. The variable was developed by assigning one point for each response which indicated the absence of the symptoms and then the scores for each item were added (total possible score was 20). A higher score of the variable indicated greater level of mental well-being. The internal consistency reliability coefficients were 0.89 (Bangladesh), 0.88 (Vietnam), and 0.89 (Ethiopia).

#### Autonomy

Financial autonomy (yes vs no) represented the women’s control over earned money. Decision-making autonomy denoted women’s involvement in household-related decisions such as major purchases; cooking; working to earn money; visiting family, friends, or relatives; healthcare visit; family planning; and care of children including child-feeding. A point was assigned if women solely or jointly (with someone else) made the decisions, then the scores were added with total possible score of 11. A higher score of the variable indicated higher level of decision-making autonomy. Employment status (employed vs not employed) was used as a measure of autonomy.

#### Social Support

The support in chores indicated receiving help from others in household tasks such as cooking, washing clothes, fetching water, fetching fuel, cleaning house and around house, taking care of the youngest child, feeding the youngest child, assisting the youngest child to bathe, and going to the market to buy food for the house. Perceived instrumental support indicated potential help with accommodation, money, and food. A point was assigned for each item if support in chores (total possible score was 8 in Bangladesh and 9 in Vietnam and Ethiopia) or perceived support (total possible score was 3) was reported, followed by addition of the scores. A higher score denoted higher level of support for both social support variables.

### Cross-Context Equivalence

Equivalence, also called invariance, helps to demonstrate that measures are consistent across contexts. Four types of equivalence are construct, item, measurement, and scalar equivalence. Construct equivalence indicates same construct is measured across contexts. In item equivalence, each item is perceived and interpretable in the same way across contexts. Item equivalence is observed when items are equally relevant and acceptable across contexts (Herdman et al. 1998). Measurement equivalence denotes construct, item, and unit are same across contexts. Measurement equivalence means that differences in one context are comparable to differences in another context. Scalar equivalence is stronger than measurement equivalence because the definition of zero is the same across contexts (Frongillo et al. 2019).

### Statistical Analysis

Statistical analysis was conducted using Stata version 14. Descriptive statistics were presented in mean and standard deviation (SD) or percentage. To address aim 1, factor analysis with varimax orthogonal rotation was performed to examine the structure of the measures of resources for care. Factor analysis helps to understand the relationships and patterns of the variables and to examine which variables can be grouped together based on their shared variance (Yong and Pearce 2013). The orthogonal rotation produces statistically uncorrelated factors with simple structure (Costello and Osborne 2005; Ford et al. 1986). The analysis included ten variables which represented the constructs of resources for care. We examined the lowest to highest number of factors until we found the most interpretable solution (Ford et al. 1986). The retention of the factors was based on the eigenvalue and scree test (Ford et al. 1986).

To evaluate equivalence for aim 2, we used the Stata *sem* command assuming one factor for each country. We ran three models with: all free parameters, measurement equivalence (i.e., metric invariance), and scalar equivalence (i.e., strong invariance). In the first model, having all parameters varying freely helps to observe the effect of not constraining factor loadings. In the second model, the factor loadings were constrained to be equal across countries. In the third model, the intercepts were also constrained to be equal across countries (Ender 2013). We used the Akaike information criterion (AIC) to assess the performance of models; smaller AIC means better fit (Fabozzi et al. 2014).

## Results

Education level was highest in Vietnam, followed by Bangladesh, and then Ethiopia (Table 1). The mean knowledge scores were 9.76, 9.01, and 8.88 in Bangladesh, Vietnam, and Ethiopia, respectively. Height was highest in Ethiopia (157 cm or 61.8 in), followed by Vietnam (153 cm or 60.2 in) and Bangladesh (151 cm or 59.4 in). Body mass index was about 20 kg/m^2^ in all three countries. Mental well-being was highest in Vietnam (mean 15.1), followed by Ethiopia (mean 14.2) and Bangladesh (mean 13.1). Financial autonomy was highest in Vietnam (73.0%) and was only 8.28% and 4.70% in Ethiopia and Bangladesh, respectively. Decision-making autonomy had a mean of about 9 in Vietnam and Ethiopia and had a slightly lower mean in Bangladesh (8.33). A substantial percentage of women were employed in Vietnam (89.2%), with lower percentage in Ethiopia (48.2%) and especially Bangladesh (6.20%). Support in chores had means of 4.48, 4.73, and 3.13 in Bangladesh, Vietnam, and Ethiopia, respectively. Perceived instrumental support was highest in Bangladesh, with the mean of 2.73, followed by Vietnam (mean 2.52) and then Ethiopia (mean 1.09).

**Table 1:** Maternal resources for care in Bangladesh, Vietnam, and Ethiopia

Resources for care	Percent or mean ± SD		
	Bangladesh (n = 4400)	Vietnam (n = 4029)	Ethiopia (n = 2746)
Years of schooling	4.82 ± 3.71	8.99 ± 3.46	1.56 ± 2.62
Knowledge (Range: B = 0–22; V and E = 0–23)	9.76 ± 2.24	9.01 ± 3.08	8.88 ± 2.97
Height, centimeters	151 ± 5.51	153 ± 5.26	157 ± 6.04
Body mass index	20.4 ± 3.18	20.1 ± 2.53	20.0 ± 2.17
Mental well-being (Range: 0–20)	13.1 ± 5.19	15.1 ± 4.50	14.2 ± 4.96
Financial autonomy, %	4.70	73.0	8.28
Decision-making (Range:0–11)	8.33 ± 2.69	9.06 ± 2.03	9.12 ± 2.59
Employed, %	6.20	89.2	48.2
Support in chores (Range: B = 0–8; V and E = 0–9)	4.48 ± 3.04	4.73 ± 3.09	3.13 ± 3.47
Perceived support (Range: 0–3)	2.73 ± 0.761	2.52 ± 0.809	1.09 ± 1.22

*B* Bangladesh, *V* Vietnam, *E* Ethiopia

A two-factor rotated model best explained the structure of resources for care in all three countries (Table 2). In all three countries, the first factor was associated with financial autonomy (factor loadings: Bangladesh 0.898, Vietnam 0.724, Ethiopia 0.413) and being employed (factor loadings: Bangladesh 0.897, Vietnam 0.646, Ethiopia 0.508). The second factor was associated with education (factor loadings: Bangladesh 0.511, Vietnam 0.539, Ethiopia 0.417) and knowledge (factor loadings: Bangladesh 0.451, Vietnam 0.539, Ethiopia 0.368). Both first (factor loading 0.326) and second factors (factor loading 0.318) were associated with decision-making in Vietnam, and the first factor (factor loading 0.316) was associated with decision-making in Ethiopia. In Bangladesh, the second factor was also associated with body mass index, mental well-being, and perceived support with the factor loadings of 0.323, 0.329, and 0.288, respectively. In Vietnam, the second factor was associated with mental well-being and perceived instrumental support, with factor-loadings of 0.266 and 0.306, respectively.

**Table 2:** Rotated factor loadings for two-factor models of maternal resources for care in Bangladesh, Vietnam, and Ethiopia

Resources for care	Bangladesh (n = 4396)		Vietnam (n = 4022)		Ethiopia (n = 2740)	
	Factor 1	Factor 2	Factor 1	Factor 2	Factor 1	Factor 2
Education	−0.0342	0.511	0.0385	0.539	−0.00770	0.417
Knowledge	−0.0248	0.451	0.0644	0.539	0.0897	0.368
Height	−0.0271	0.130	−0.0154	0.0874	−0.0829	0.183
Body mass index	−0.0117	0.323	−0.0665	0.0599	0.0324	−0.0117
Mental well-being	−0.0297	0.329	−0.0490	0.266	0.153	0.166
Decision-making	0.0553	0.108	0.326	0.318	0.316	0.0683
Financial autonomy	0.898	0.000400	0.724	0.0998	0.413	0.121
Employed	0.897	−0.0214	0.646	−0.0641	0.508	−0.0308
Support in chores	0.0362	0.0908	−0.00380	0.134	0.108	0.00650
Perceived support	−0.0509	0.288	0.0787	0.306	0.219	0.206

Regarding equivalence, the models with all free parameters, constrained loadings, and constrained both loadings and intercepts had, respectively, AIC of 448,091, 448,443, and 467,732 (with corresponding 87, 69, and 51 degrees of freedom). The AIC for the first two models were similar and both were smaller than that for the third model. These results are consistent with there being measurement equivalence but not scalar equivalence across countries.

## Discussion

The overall structure of resources for care was similar across countries. In all three countries, financial autonomy and employment loaded on the same factor. Decision-making autonomy loaded along with these two variables in Vietnam and Ethiopia. Employment, financial autonomy, and decision-making are regarded as attributes that help individuals to control the surroundings and make life choices (Engle 1999). In this study, loading of financial autonomy and employment in the same factor was expected because, to be financially autonomous, women needed to meet two criteria which were being employed and having authority to use the earned money. Additionally, women who are employed have greater influence in various domains of household decision-making such as seeking healthcare, household purchases, and visiting family and friends (Engle et al. 1999). In contrast, employment may reduce availability of time to care for family including children (Engle et al. 1999); the surveys used for this study lacked information on availability of time.

Education and knowledge loaded on the same factor in all three countries. Education and knowledge are placed in the same category in the extended model of care (Engle et al. 1999). Formal education transmits health and nutrition-related information directly and provides skills to get knowledge from mass-media and improves confidence which could enhance knowledge (Engle et al. 1999). Nevertheless, education may not necessarily improve all aspects of knowledge (Reich 2005). Knowledge could be influenced by attributes other than formal educational attainment such as culture, public campaigns, and health and nutrition programs (Reich 2005). Improving knowledge among women with low educational attainment can be an effective strategy to improve children’s health. For instance, a study from Pakistan found that improving maternal knowledge about vaccines has potential to increase child immunizations rate among populations with low- income and low- literacy (Owais et al. 2011).

In Bangladesh, body mass index, mental well-being, and perceived instrumental support loaded on the second factor, but the factor loadings were not high. In Vietnam, mental well-being and perceived instrumental support loaded on the second factor but the factor loadings were not high. In all three countries, height and support in chores did not load strongly in any of the factors. These findings are consistent with height, body mass index, mental well-being, support in chores, and perceived support being distinct resources for care. Although height and body mass index are measures of physical health, height reflects the nutritional condition of women in utero, early years of life, and across the life course; in contrast, body mass index reflects the more immediate nutritional status (Subramanian et al. 2009). Mental well-being helps to translate acquired information and skills into practice and has been linked with the provision of quality care to the children (Engle et al. 1999). Although mental health may be related to other resources like physical health (Jacka et al. 2012), assessment of mental well-being as in this study may provide information on psychosomatic difficulties women are facing (Harpham et al. 2005). In this study, perceived support was associated with second factor but support in chores was not associated with any of the factors. Previous studies have also separated perceived and received social support in terms of conceptualization and measurement (Uchino 2009). The weak relationship between perceived and received social support has been reported in previous research (Haber et al. 2007). Perceived support is reported to have more consistent associations with health outcomes than received support (Uchino 2009). In this study, support related to household chores was used to reflect received support, although other types or dimensions of received social support exist. Additionally, we were unable to investigate the relationship of support in chores with reasonable workload or availability of time due to the lack of data on workload or time. Women are typically involved in household work, and support in chores may reduce workload of women and increase availability of time (Engle 1999; Engle et al. 1999). Future research is needed to investigate the relationships of social support with reasonable workload and availability of time and other resources for care.

The measures of resources for care were measurement equivalent across countries, meaning that constructs, items, and units were similar across countries (Frongillo et al. 2019). Measurement equivalence means that a difference in one context is comparable to the same difference in another context. Ensuring measurement equivalence is essential in cross-national comparative research (Kankaraš and Moors 2010). Our findings suggest that the measures of resources for care can be used to examine resources for care in low- and middle-income countries. Our study did not demonstrate scalar equivalence across countries. Scalar equivalence, which means that zero is defined the same across contexts, is harder to achieve than other types of equivalence (Frongillo et al. 2019; Kankaraš and Moors 2010). The lack of scalar equivalence means that prevalence estimates are not necessarily comparable across contexts.

Although the structure of resources for care was similar and equivalent across countries, resources for care can be influenced by context. For example, a study conducted in India and Pakistan reported that geographic region played a strong and consistent role in determining the levels and patterns of women’s autonomy, whereas religion and nationality played a modest and inconsistent role (Jejeebhoy and Sathar 2001). Additionally, being employed and financially autonomous may be insufficient for improving decision-making autonomy in some settings. This explanation is supported by a study from Bangladesh which found that gender inequalities in seeking healthcare was present even after socio-economic interventions (Ahmed et al. 2000). We also found that decision-making autonomy, financial autonomy, and employment loaded on the same factor in Vietnam and Ethiopia but not in Bangladesh.

The strengths of this study were large sample sizes, inclusion of three socio-culturally diverse countries, and strong theoretical grounding (Engle et al. 1999). The data collection procedures decreased the chances of biases for self-reported data by carefully training data collectors, closely supervising all data collection steps, and ensuring privacy when conducting the surveys so women could be comfortable to answer all questions. Height and weight were measured using standard anthropometric techniques. The instrument used to collect data on mental well-being was validated for low- and middle-income settings (WHO 1994). The limitations are lack of information on workload, use of cross-sectional data and self-reported measures, and limited generalizability of the findings to the high-income countries.

In conclusion, we examined the factor structure and equivalence of the measures of resources for care across three countries. In all three countries, a two-factor rotated model best explained the structure, and the resources for care were structurally similar. The resources for care variables also were measurement equivalent across the countries. The study findings support the use of measures of resources for care to assess and compare resources for care in low- and middle-income countries. Additional work examining the structure and cross-context equivalence of resources for care in other settings is warranted.
