# Correction to: Factor Structure and Equivalence of Maternal Resources for Care in Bangladesh, Vietnam, and Ethiopia

**DOI:** 10.1007/s10995-021-03122-6

**Published:** 2021-04-22

**Authors:** Sulochana Basnet, Edward A. Frongillo, Phuong Hong Nguyen, Spencer Moore, Mandana Arabi

**Affiliations:** 1 Department of Health Promotion, University of South Carolina, Education, and Behavior, 915 Greene Street, Discovery I, Columbia, SC 29208-4005 USA; 2 Department of Health Promotion, Education, and Behavior, University of South Carolina, Columbia, SC USA; 3 International Food Policy Research Institute, Washington, DC USA; 4 Global Technical Services, Nutrition International, Ottawa, ON Canada

## Correction to: Maternal and Child Health Journal 10.1007/s10995-020-03100-4

The article “Factor Structure and Equivalence of Maternal Resources for Care in Bangladesh, Vietnam, and Ethiopia”, written by Sulochana Basnet, Edward A. Frongillo, Phuong Hong Nguyen, Spencer Moore and Mandana Arabi, was originally published electronically on the publisher's internet portal on 25 February 2021 without open access. With the author(s)' decision to opt for Open Choice the copyright of the article changed on 26 March 2021 to © The Author(s) 2021 and the article is forthwith distributed under a Creative Commons Attribution 4.0 Inter-national License, which permits use, sharing, adaptation, distribution and reproduction in any medium or format, as long as you give appropriate credit to the original author(s) and the source, provide a link to the Creative Commons licence, and indicate if changes were made. The images or other third party material in this article are included in the article's Creative Commons licence, unless indicated otherwise in a credit line to the material. If material is not included in the article's Creative Commons licence and your intended use is not permitted by statutory regulation or exceeds the permitted use, you will need to obtain per-mission directly from the copyright holder. To view a copy of this licence, visit https://creativecommons.org/licen ses/by/4.0. The original article has been corrected.

